# In vitro activity of encapsulated lactic acid bacteria on aflatoxin production and growth of *Aspergillus* Spp

**DOI:** 10.1002/fsn3.2015

**Published:** 2021-01-19

**Authors:** Rouhollah mohammadi, Sepideh Abbaszadeh, Aghil Sharifzadeh, Mojtaba Sepandi, Maryam Taghdir, Najmeh Youseftabar Miri, Karim Parastouei

**Affiliations:** ^1^ Health Research Center Life style institute Baqiyatallah University of Medical Sciences Tehran Iran; ^2^ Department of Nutrition and Food Hygiene Faculty of Health Baqiyatallah University of Medical Sciences Tehran Iran; ^3^ Department of Microbiology and Immunology, Faculty of Veterinary Medicine University of Tehran Tehran Iran; ^4^ Department of Food Science Faculty of Agriculture Ferdowsi University Mashhad Iran

**Keywords:** *Aflatoxin*, *Antifungal activity*, *Aspergillus*, Lactic acid bacteria

## Abstract

This study aimed to investigate the potential ability of simultaneously used *L. acidophilus(LA‐5), L.rhamnosus(LGG),* and *L.casei(LC‐01)* in encapsulated (E) and nonencapsulated (NE) forms in mycelial growth of *Aspergillus* spp and aflatoxin production by *A. flavus*. In order to assess the zone of fungal growth inhibition by E and NE lactic acid bacteria, the agar well diffusion method was applied. Quantification of aflatoxin was performed using a high‐performance liquid chromatography technique. Lactic acid bacteria exhibited high antifungal activity and significantly reduced AFB1, AFB2, AFG1, and AFG2 production in both E and NE forms compared to the control group. The percentage of reduction in total AFs production in treated samples with E and NE lactic acid bacteria was 94.1% and 95.5%, respectively. These results suggested that simultaneously used lactic acid bacteria in E and NE forms can prevent growth and decrease aflatoxin production of toxigenic aspergilla.

## Graphical abstract

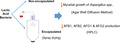

## 1 INTRODUCTION

Fungal species are regarded as common contaminants of food, and they negatively affect the food quality and shelf‐life. Around the world, fungal spoilage of food imposes vast economic losses annually. It has been stated that almost 5% –10% of food across the world and 50% of fruits and vegetables are lost yearly in tropical zones owing to fungal spoilage (Faizan et al., 2019). Many fungal species may produce mycotoxins (fungal toxic secondary metabolites), including aflatoxins, ochratoxins, and fumonisins, which lead to an extensive threat to food safety (Milicevic et al., 2016). In food products, Aspergillus is one of the most common mycotoxigenic fungal species, and aflatoxins (AFs), among mycotoxins, are the most toxic. The most carcinogenic AF is Aflatoxin B_1_ (AFB_1_) (Ahlberg et al., 2015), and it causes chronic and acute intoxication in human being (Shetty et al., 2007). AFs are potent toxic secondary metabolites produced mainly by *A. flavus and A. parasiticus*. The food contamination with AFs causes severe financial losses and health problems (Furukawa & Sakuda, 2019). Aflatoxicosis is the acute form of exposure to AFs of severe hepatotoxicity cases; chronic exposure is associated with hepatocellular carcinoma, immunological suppression, and effects on nutritional status (Strosnider et al., 2006).

Chemical, biological, and physical methods such as oxidation, alkalization, medicinal plants, nanoparticles, and irradiation have been reported to eradicate, inactivate, and inhibit the growth of fungal spieces and the production of mycotoxins (Tola & Kebede, 2016). High costs, low nutritional value, undesirable organoleptic characteristics, and unknown health effects were the adverse points of the previous methods; however, some indigenous microorganisms, which traditionally are used in foods, have potential in the prevention of health adverse effects of mycotoxin (Bhat et al., 2010; Di Natale et al., 2009).

Studies showed that Lactic acid bacteria (LAB) are suitable for preventing fungal growth and prolong the shelf‐life of food products (Faizan et al., 2019; Dalié et al., 2010). Organic acids, diacetyl, fatty acids, bioactive antimycotic peptides, bacteriocins, carboxylic acids, lactones, hydrogen peroxide, reuterin, and alcohols are the reported antifungal compounds produced by several LABs (Crowley et al., 2013). Many elements have been proven to affect the viability of LAB in food, such as pH, storage temperature, together with processing conditions (Kim et al., 2008). After consumption, the viable LAB in food must survive the gastrointestinal ecosystem with estimated 0.3% bile salt concentration (Mainville et al., 2005; Shah, 2007). Consequently, it is essential to use a method to selectively protect these usefull organisms under adverse conditions without affecting their antifungal effects. Encapsulation is an operational protection of the LAB against these affected conditions (Nedovic et al., 2011). One of the most common microencapsulation techniques in the food industry is spray drying that engages with the atomization of a LAB suspension and coating material into a drying gas chamber, causing quick water evaporation. Numerous studies have reported the efficiency of this quick and less expensive method to protect microorganisms (Yonekura et al., 2014; Ilha et al., 2015; Rokka and Rantamäki, 2010; Meyer et al., 2019,; Assadpour & Jafari, 2019), which produces powders with tiny particle sizes and a smoother mouthfeel; this would allow the LAB addition to a broader range of foods (Yonekura et al., 2014). Whey proteins, as encapsulation biomaterial, increase the viability of the LAB in adverse conditions (Abd El‐Salam and El‐Shibiny, 2015). No studies have been conducted on the antifungal effects of spray‐dried LAB and its effect on AF production; thus, this study aims to evaluate the effects of LAB encapsulation with spray drying on AF production and growth of Aspergillus spp.

## 2 MATERIALS AND METHODS

### 2.1 Microorganisms and culture conditions

**Figure 1:**
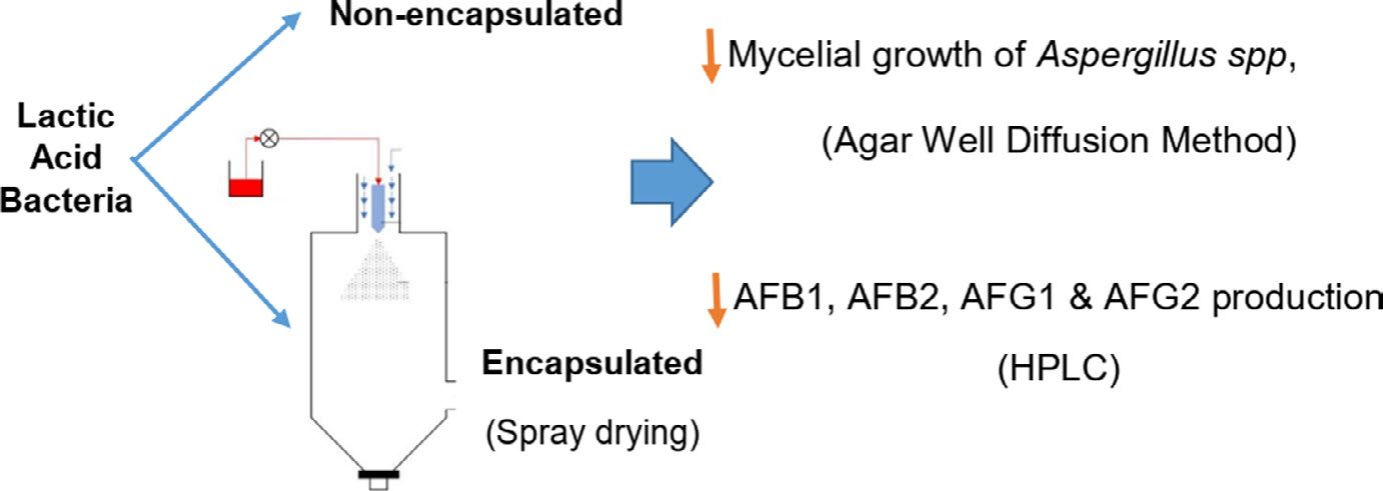
This study showed that Lactic acid bacteria exhibited antifungal activity and significantly reduced AFB1, AFB2, AFG1, and AFG2 production in both encapsulated and nonencapsulated forms compared to the control group. The results of this study suggested that simultaneously used lactic acid bacteria in E and NE forms can prevent growth and decrease aflatoxin production of toxigenic aspergilla

*L. acidophilus* (LA‐5), *L. rhamnosus* (LGG), and *L. casei* (LC‐01) were bought from CHR‐Hensen Co., Denmark. All these microorganisms were maintained in sterile 20% v/v glycerol at − 80°C before use. Bacterial colonies were kept at 4 ˚C on MRS agar plates (BioMerieux, France) for next steps. *A. niger* (PTCC 5,012), *A. flavus* (PTCC 5,004), and *A. parasiticus* (PTCC 5,286) as bread‐spoiling fungi, were purchased from the Iranian Research Organization for Science and Technology (IROST) and applied as test cultures in antifungal assays. They were maintained on Potato Dextrose Agar (PDA, Oxoid, Hampshire, UK) plates at 25 ˚C for 72 hr (Figure 1).

#### 2.1.1 Encapsulation by spray drying

**Figure 2:**
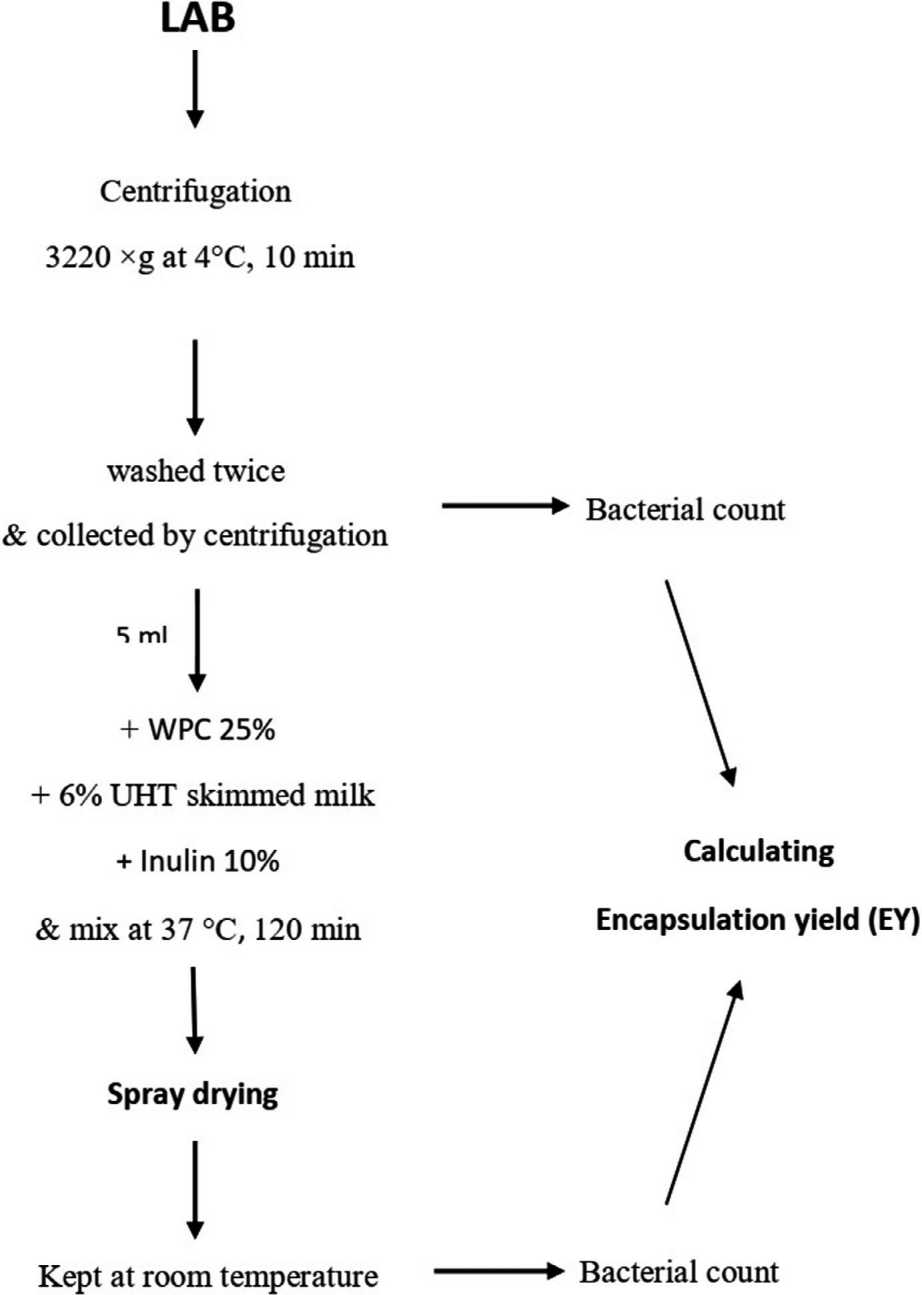
Flowchart for LAB encapsulation with spray drying method

Commercial skim milk powder (Sigma‐Aldrich, Steinhein, Germany) was used for the bacterial suspension preparation. Whey protein concentrate (WPC) (25%) (5.99% total solids and 35.28% protein) (Razavizadeh et al., 2014) and the inulin 10% (Santosa et al., 2019), as carrier agents, were provided in distilled water and sterilized for 15 min at 121°C. By centrifugation at 3,220 × g at 4°C for 10 min, the mixed bacterial cultures (LA‐5, LGG, and LC‐01 in equal proportions) were harvested, twice washed with sterile saline, and collected by centrifugation. The washed bacterial cells were resuspended in 5 ml sterile saline, and the mixed cell count was determined using the surface plate method in duplicate in MRS agar. The mixture ratio of LAB strain and the coating material was 1:9 (v/v). For encapsulation, 5 ml prepared bacterial was suspended in a sterilized premixture with 6% UHT skimmed milk. Then, the suspension was mixed at 37°C for 120 min. The process of spray drying was carried out based on a modified technique with lower temperatures and more cell viability (Fritzen‐Freire et al., 2013; Bustamante et al., 2015). A mini spray dryer (B190, Buchi, Switzerland) was applied with a 120°C air inlet temperature. Prior to being applied, encapsulating agent solution with bacterial culture was maintained at room temperature under magnetic stirring. The compressor air pressure of 0.4 MPa and a drying airflow rate of 55 m3/ h were applied (Santosa et al., 2019). After spray drying, the final powder was placed in sterile dark flasks and kept at room temperature (Figure 2).

The microcapsules (1.0 g) achieved through spray drying were suspended with 9 ml sterile normal saline and mixed for 1 min, to release the cells. After adequate dilution, the numbers of entrapped cells were measured by using surface plate count technique and encapsulation yield (EY) was computed through Equation 1:

EY (%) = (log N/ log N_0_) ×100 [1]. Where N was the number of viable entrapped cells (CFU/ml) released from the encapsulates, and N_0_ was the number of free viable cells (CFU/ml) before encapsulation.

### 2.2 Antifungal effect

After aerobic incubation for 48 hr at 37 ˚C, free mixed bacterial culture were cultured on MRS (Abbaszadeh et al., 2015). The agar well diffusion method was used to assess the inhibitory activity (Abbaszadeh et al., 2015). Each fungal suspension counted with Neubauer counting chamber and mixed with MRS agar (10^5^ conidia/ml), after cooled to 55°C, dispended on strile plates (10ml in each plate), and permitted to solidify. Wells with 5 mm diameters were then prepared with Pasteur pipettes in the center of each plate. 10 µl of warm MRS agar was poured in each well in order to cover the base of the wells. Then, 100 µl of a log‐phase culture of the free mixed LAB bacteria (10^6^ CFU/ml) was added to each well. Encapsulated LAB was suspended with saline and then added to each well. After 5‐day incubation of the plates at 30 ˚C, the growth inhibition was defined by measuring the inhibition region surrounding each agar well. On two separate times, all experiments were repeated in duplicate on each occasion. The fungal inhibition of each group against tested fungi was computed through the following Equation 2:

FI (%) = (IR/GR) × 100; [2].

FI: fungal inhibition; IR: inhibition radius; GR: growth radius.

### 2.3 Fungal dry weight (FDW) determination

Based on the method of *Rasooli* and *Razzaghi‐Abyaneh,* fungal dry weight was computed. The fungal mycelia were filtered through Whatman filter no. 1, and washed carefully with distilled water. The mycelial mats were located on preweighed Petri plates and allowed to dry for 6 hr at 60ºC and then kept for 18 hr at 40ºC. The petri plates containing dry mycelia were then weighted. This procedure was performed for both LAB treated and control groups.

### 2.4 Effect on Aflatoxin production

The fungal spore suspension was provided with the method of Fan and Chen (1999). From PDA solid cultures in a sterile phosphate buffer solution with Tween 80 (0.05%), spores of *A. flavus* incubated at 25°C were collected. A flamed wire loop was used to loosen the spores. By filtration through sterile cheesecloth, mycelial debris was removed. It was diluted further to achieve a final spore suspension with about 1.5 × 10^7^ spores/ml determined with a Neubauer counting chamber; for all experiments, the spread plate method on PDA plates was applied.

The effect of *lactobacilli* on AF production by *A. flavus* was detected through inoculating 1 ml bacterial suspension with 1 McFarland concentration (E and NE) in yeast extract sucrose broth (YESB) supplemented with fungal spores (10^7^ spores/ml), followed by incubation for 10 days at 28°C and 100 rpm. Plain YESB media and YESB media supplemented with known fungal spores were also incubated as negative and positive controls, respectively, with no bacterial inoculation. Medium with lactobacilli and fungus, after incubation, was filtered through Whatman filter paper 1#, extracted and analyzed by HPLC, and then compared with controls (Huang et al., 2017).

### 2.5 AFs extraction

AFs were extracted from YESB based on the AOAC standard technique with certain modifications. Concisely, the AFs were extracted from a 50 ml YESB with 300 ml of a solvent mixture of methanol, n‐hexane (100 ml), and water (80:20,v/v) and mixing for 3 min vigorously in a high‐speed blender. A fluted filter paper was used to filter the extract, and 10 ml of filtrate was diluted and mixed with a solution of phosphate buffer (1:6,v/v). The mixture was injected to an immune‐affinity column with specific antibodies of AFs B1, B2, G1, and G2. The AFs were eluted with methanol from immune‐affinity columns into acid‐washed vials, and finally, 20 µl was applied into HPLC (Hassan & Habibi, 2011).

### 2.6 Determination of AFs

The HPLC apparatus was applied to determine AFs. The HPLC setup comprised an Agilent 1,200 Series quaternary pump and a standard autosampler, using a A 1,200 series fluorescence detector with Agilent Technologies (Palo Alto, USA). To manage data, Agilent's ChemStation software was employed. Combinations of three reagents were involved in the mobile phase. AF level determination in the sample extracts was done by isocratic reversed‐phase liquid chromatography with Symmetry^®^ C18 column with a 5µm particle diameter, 100 A° pore diameter with matching guard column (Waters Corporation, Milford, USA) and a Kobra cell. For derivatization and fluorescence detector, excitation and emission wavelengths were 362 and 440 nm, respectively. Methanol, acetonitrile, and water (20:20:60 v/v) with 119 mg of potassium bromide, 100 µl of 65% Nitric acid were the mobile phase and filtered through a Millipore 0.22 µm membrane filter before use. At the same fluorescence emission wavelength in a single run, AFs elute in the order of G_2_, G_1_, B_2_, and B_1_(Hassan & Habibi, 2011).

### 2.7 Statistical analysis

The tests were carried out in triplicate. In this research, the collected data were expressed as mean ± standard deviation; they were subjected to a one‐way analysis of variance (ANOVA) and dependent samples *t* test. Tukey's test was applied to perform multiple comparisons. Statistical significance was set at *p* < .05. SPSS Version 16.0.1 was applied to perform all analyses (SPSS Inc., Chicago, USA).

## 3 RESULTS AND DISCUSSION

### 3.1 Encapsulation yield (EY)

The average EY obtained in the current study was 96.8 ± 0.8% that is similar to those reported in previous studies using skim milk and cheese whey for spray drying of *L. paracasei* with 93.12% survival rate (Ilha et al., 2015). Lower EY results were found by Maciel et al., with an average EY of 76.58% for spray‐dried *L. acidophilus* La‐5 microcapsules with reconstituted sweet whey. According to previous studies, the survival of spray‐dried LAB depends on the outlet temperature of spray dryer, the types and concentrations of the encapsulating agent, as well as considering that generally, cell culture from a stationary phase survived better than the log‐phase culture (Corcoran et al., 2004). Although spray drying causes some physical injuries to the microcapsules, the bacterial cells release, and heat generation, as shown in this study, the spray‐dried method with proper liquid feed solution can have an acceptable EY. Whey protein is a favorable biomaterial for probiotic encapsulation due to its effect on bacterial entrapment and prevention of cells overheat in high temperatures (Doherty et al., 2010).

### 3.2 Antifungal effect

**Figure 3:**
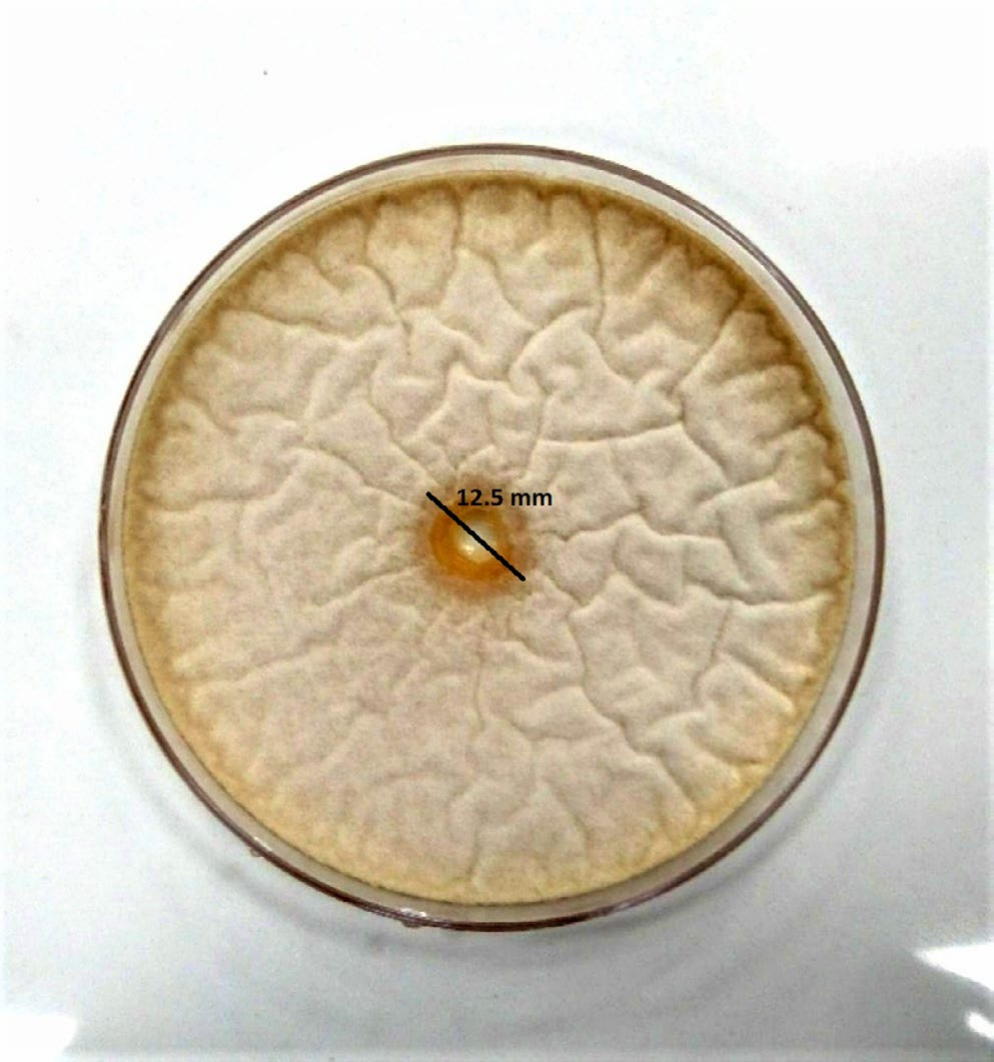
Agar well diffusion assay for the determination of antifungal activity of LAB. Black line indicate the zone of inhibition (mm)

Antifungal activity of encapsulated (E) and nonencapsulated (NE) LAB was examined on three important bread spoilage fungal species with the well diffusion method (Figure 3). The amounts of FI showed that both E and NE bacteria inhibited fungal growth (Table 1). Although the antifungal activity of NE bacteria was a bit more than E, the differences were not significant (*p* > .05); it showed that spray drying encapsulation could not significantly reduce the LAB inhibitory effect. Although previous studies showed several LAB as potential bio‐preservative agents with a broad antifungal spectrum (Faizan et al., 2019; Kabak & Var, 2004; Bueno et al., 2006), no studies conducted on the antifungal effect of spray‐dried encapsulated LAB bacteria. Studies showed that LAB prevents the growth of fungi through producing metabolites, including organic acids, cyclic peptides, reuterin, and hydrogen peroxide (Sadiq et al., 2019). Guimaraes et al. (2018) reported 50% inhibition growth of *Penicillium nordicum* with LAB supernatant containing organic acids.

**Table 1:** Fungal inhibition (%) of encapsulated and nonencapsulated lactic acid bacteria using well diffusion method

	FI (%) of lactic acid bacteria	
	Encapsulated	Nonencapsulated
*A. flavus*	22.5 ± 1.4 ^Aa^	26.1 ± 2.5 ^Aa^
*A. niger*	23.6 ± 2.7 ^Aa^	26.1 ± 0.9 ^Aa^
*A. parasiticus*	26.1 ± 3.5 ^Aa^	36.7 ± 3.8^Ab^

Note

Values are mean ± *SD*. Values with the same lowercase letter in the same column are not significantly different (*p* > .05). Values with the same capital letter in the same raw are not significantly different (*p* > .05).

### 3.3 Aflatoxin production

Various studies have shown that inhibiting the growth of mycotoxin‐producing fungi is one of the best ways to prevent and reduce food contaminations with a mycotoxin (Blagojev et al., 2012). Along these lines, the AF binding ability of lactobacilli is a suitable biological way to prevent and control fungal food poisoning (Haskard et al., 2001; Peltonen et al., 2001). Previous investigations have shown that suspension inoculation containing a mixture of different species of lactobacillus inhibits mycotoxin biosynthesis in *Aspergillus* and *Penicillium* species (Gourama & Bullerman, 1995; Karunaratne et al., 1990). Thus far, no specific study has been performed concerning the effect of encapsulated LAB on their ability to inhibit AF production. In the present study, E and NE lactic acid bacteria presented the highest antifungal activity in the previous studies were simultaneously used against mycelium development and AF production by a toxigenic strain of *A. flavus* (Abbaszadeh et al., 2015). The mycelium production and the concentration of AFB1, AFB2, AFG1, AFG2, as well as total AFs detected in treated samples with E and NE‐LAB, were analyzed (Table 2). According to the results, *L. rhamnosus* (LGG), *L. acidophilus* (LA‐5), and *L. casei* (LC‐01) significantly reduced AFB1, AFB2, AFG1, and AFG2 production in both E and NE forms compared to the control group (*p* < .05). The percentage of reduction in total AFs production in treated samples with E and NE‐LAB was 94.1% and 95.5%, respectively. In agreement with the present work's results, previous studies demonstrated that lactobacilli inhibit AF production, as well as the growth of Aspergillus spp (Chang & Kim, 2007; Gerbaldoet al., 2012; Huang et al., 2017).

**Table 2:** Inhibitory effects of encapsulated and nonencapsulated lactic acid bacteria on aflatoxin production and mycelium dry weight (MDW)

Lactic Acid Bacteria	Aflatoxins (ppb)					
	B1	B2	G1	G2	Total AFs	MDW (mg/l)
Encapsulated bacteria	1,485 ± 18.4 ^A^	994.2 ± 5.4 ^A^	608.8 ± 11.5^A^	1,197 ± 4.2 ^A^	4,285 ± 31.1 ^A^	70.6 ± 0.6 ^AB^
Nonencapsulated bacteria	1,202.5 ± 6.4 ^B^	722.5 ± 17.7 ^B^	414.5 ± 7.8 ^B^	958 ± 4.2 ^B^	3,297.5 ± 0.71 ^B^	67.6 ± 0.6 ^A^
Control (without bacteria)	38,599.5 ± 14.8 ^C^	18,905 ± 63.6 ^C^	9,474 ± 36.8 ^C^	5,925.5 ± 7.8^C^	72,904 ± 19.8 ^C^	72.6 ± 1.3 ^B^

Note

Values are mean ± *SD*. Values with the same letter in the same column are not significantly different (*p* > .05).

The percentage of reduction in AF production was 99.2% and 95% by *L.casei* and *Bacillus subtilis*, respectively (Chang & Kim, 2007; Kimura & Hirano, 1988). In the current study, in the NE group, the reduction in AF production was greater than the E group; however, this difference was not significant. Lactobacillus binding to AFs is a possible mechanism of AF reduction by lactobacillus (Chlebicz and Śliżewska, 2020, Hashemi & Amiri, 2020), and also, it is a reason for the greater effects of NE lactobacillus than that of encapsulated, although this difference is not significant, can be the decrease in binding due to encapsulation.

The encapsulated bacteria, after the treatment period, did not show any significant difference in mycelium dry weight of *A. flavus* compared to the NE and control group.

However, the NE group significantly decreased the mycelium dry weight of mycotic mass compared to the control group. The results of nonencapsulated bacteria are in line with the study of Chang in which *L. casei* inhibited the growth and mycelium dry weight *A. flavus* (Chang & Kim, 2007).

## 4 CONCLUSION

In the present study, antifungal and anti‐aflatoxin effects of encapsulated LAB bacteria are tested for the first time, and results show that spray drying is a useful method for large‐scale preparation of encapsulated bacteria, aiming antifungal properties in food systems. This study is currently in progress to examine the effectiveness of such encapsulated bacteria on fungal contaminated bread.
